# Diverse transposable element landscapes in pathogenic and nonpathogenic yeast models: the value of a comparative perspective

**DOI:** 10.1186/s13100-020-00215-x

**Published:** 2020-04-21

**Authors:** Patrick H. Maxwell

**Affiliations:** Biology Department, Siena College, Loudonville, NY USA

**Keywords:** *Candida albicans*, *Cryptococcus neoformans*, *Saccharomyces cerevisiae*, *Schizosaccharomyces pombe*, Retrotransposon, Transposable element, Tf1, Tf2, Ty1, Ty3

## Abstract

Genomics and other large-scale analyses have drawn increasing attention to the potential impacts of transposable elements (TEs) on their host genomes. However, it remains challenging to transition from identifying potential roles to clearly demonstrating the level of impact TEs have on genome evolution and possible functions that they contribute to their host organisms. I summarize TE content and distribution in four well-characterized yeast model systems in this review: the pathogens *Candida albicans* and *Cryptococcus neoformans*, and the nonpathogenic species *Saccharomyces cerevisiae* and *Schizosaccharomyces pombe*. I compare and contrast their TE landscapes to their lifecycles, genomic features, as well as the presence and nature of RNA interference pathways in each species to highlight the valuable diversity represented by these models for functional studies of TEs. I then review the regulation and impacts of the Ty1 and Ty3 retrotransposons from *Saccharomyces cerevisiae* and Tf1 and Tf2 retrotransposons from *Schizosaccharomyces pombe* to emphasize parallels and distinctions between these well-studied elements. I propose that further characterization of TEs in the pathogenic yeasts would enable this set of four yeast species to become an excellent set of models for comparative functional studies to address outstanding questions about TE-host relationships.

## Background

**Fig. 1:**
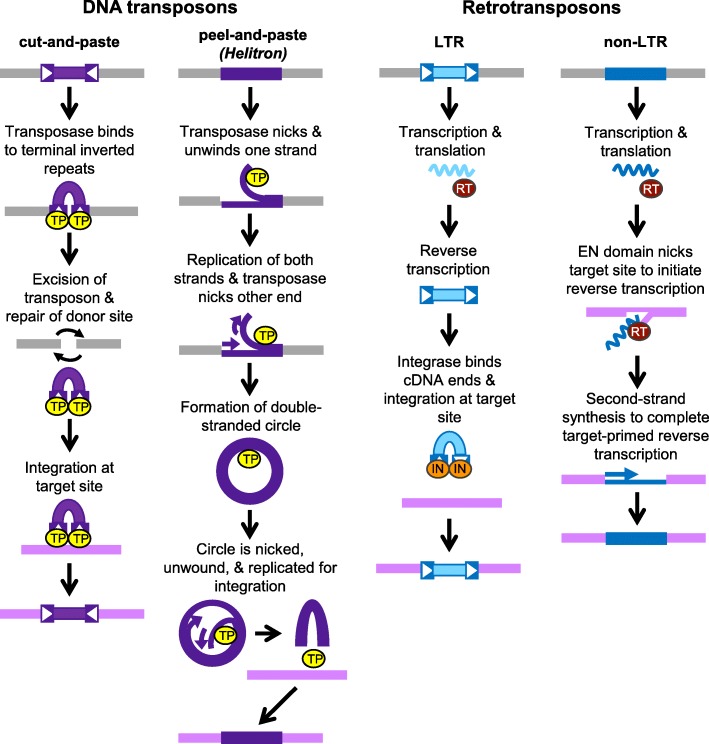
Comparison of transposition cycles of four subclasses of transposable elements (TEs). Simplified representations of major steps in the transposition cycles of two subclasses of DNA transposons (cut-and-paste and peel-and-paste) and two subclasses of retrotransposons (long terminal repeat (LTR) and non-LTR) are shown. Light gray and lavender lines represent donor and target genomic sites, respectively. Boxed arrowheads indicate terminal inverted repeats (cut-and-paste) or LTRs. Curved black arrows indicate repair of the donor site after transposon excision, small purple or blue arrows represent new DNA synthesis, and wavy lines represent RNA. TP- transposase (DDE-type for cut-and-paste or HUH nuclease/helicase-type for peel-and-paste), RT- reverse transcriptase, IN- integrase, EN- endonuclease domain.

Transposable elements (TEs) are a diverse set of genetic elements that can move to new sites in genomes and substantially contribute to genotypic and phenotypic variation. They are divided into two main classes, followed by subclasses, superfamilies, and families based on their sequence structures and mechanisms of mobility, or transposition [1]. Mobility of Class 1 elements, known as retrotransposons, occurs through reverse transcription of an RNA into a complementary DNA (cDNA) that is inserted at a new genomic site, which is referred to as a “copy-and-paste” mechanism (Fig. 1). Two example subclasses are long terminal repeat (LTR) retrotransposons, with the four superfamilies Ty1/*copia* (Pseudoviridae), Ty3/*gypsy* (Metaviridae), BEL, and endogenous retroviruses, and non-LTR retrotransposons, with approximately 30 superfamilies, such as CRE, I, *jockey*, L1, and R2 [1]. LTR retrotransposons reverse transcribe cDNA prior to integration by DDE-type integrases, while non-LTR retrotransposons nick target sites using endonuclease domains to initiate reverse transcription (Fig. 1) [1, 2]. Mobility of Class 2 elements, known as DNA transposons, involves DDE transposase proteins that excise DNA copies of elements from donor sites and integrate them at new genomic sites, a “cut-and-paste” mechanism, for approximately 20 superfamilies, such as *EnSpm*, *Harbinger*, *Mariner*/Tc1, and MULE (*Mutator*-like elements) (Fig. 1) [1, 2]. Mobility of the *Crypton* superfamily involves tyrosine recombinases and possible circular DNA intermediates, while *Helitron* mobility involves transposases with HUH nuclease and helicase activities that nick and unwind one transposon strand, which is replicated to form a double-stranded circular DNA that is used to integrate a copy at a new site (“peel-and-paste”, Fig. 1) [2–4].

TEs are widely known for causing mutations when they are mobile, but they also contribute to chromosomal rearrangements, and have transcriptional control sequences that can affect expression of neighboring genes [2]. Unrestricted activity of a mutagenic agent is typically deleterious, so organisms have many layers of defense that restrict TE mobility at various stages of their complex transposition cycles [2]. It has long been discussed that activation of TEs by stress could produce random genetic variation that gives rise to occasional genotypes better adapted to the relevant stress. Many TEs are activated by particular stresses, and there is some evidence that their transcription or transposition can produce beneficial mutations or changes in gene expression for adaptation to stress [5]. An increasing number of studies, particularly large-scale genomic, transcriptomic, and epigenomic studies, are providing evidence that TEs dispersed throughout genomes provide cis-regulatory sequences and transcriptional start sites for expression of neighboring genes [6]. There are also observations consistent with particular groups of TEs with common cis-regulatory sequences dispersed at various genomic sites contributing to the evolution of gene regulatory networks coordinately controlling large numbers of genes [6]. TEs are also now being analyzed on a massive scale. For instance, a recent study of DNA transposons in 1730 fungal genomes shows that DNA transposon content is correlated with fungal lifestyles (such as soil-living, associated with plants or animals), and increased numbers of DNA transposons but fewer functional copies are present in species with RNA interference defense systems [7]. A second study of total TE content in 625 fungal genomes by the same group reports that the number of TEs in genes is correlated with fungal lifestyles, and differences in clustering of TEs in genomic regions is correlated with their potential for being active elements [8].

The types of studies just discussed are very important and exciting, and are leading to increased interest in studying TEs. However, they must be thoughtfully considered and presented, because they raise exciting possibilities, but in many cases additional functional studies are needed to verify these possibilities [2, 5, 6]. For instance, TEs differ in whether they are activated or inhibited by particular stresses, how they affect expression of neighboring genes, and evidence of negative impacts of TEs during stress have been reported [5]. Biochemical activities consistent with cellular functions for TEs do not guarantee that TEs have those relevant functions, pointing to the need for follow-up functional studies. For example, functional analyses of TE enhancer sequences expected to regulate gene expression in mouse embryonic stem cells based on transcriptomic and epigenomic data show that only a small subset of the enhancers tested had substantial effects on gene expression [9]. Furthermore, one of the large-scale studies of fungi mentioned earlier shows that nearly all TEs analyzed are likely to be experiencing neutral evolution [8]. Overall, there is a growing discussion of the need to experimentally examine the potentially significant impacts of TEs on their hosts [2, 5, 6].

I propose that the model yeast species discussed in this review represent excellent systems for comparative functional analyses to develop a more sophisticated understanding of the regulation and impacts of TEs. These four yeasts – the human pathogens *Candida albicans* and *Cryptococcus neoformans*, and the nonpathogenic model organisms *Saccharomyces cerevisiae* and *Schizosaccharomyces pombe* – are all well-developed model species with diverse lifestyles and genomic features that may have influenced the evolution of TE-host relationships. I will summarize TE content and distribution, genomic features, lifestyles, and presence of RNA interference pathways in each species to highlight similarities and differences for considering comparative studies. Such studies comparing most/all TEs in a genome, as well as differences in TE regulation/impact between multiple strains of each species, or between the four diverse species has great potential to address many questions about TE evolution and impact on host organisms.

### Genomic features of the four yeasts

Yeasts in general have small gene-dense genomes with relatively low TE content that can facilitate manipulation and evaluation of a large proportion of the total TEs in a genome. Genomic features data are shown for reference genomes for two major varieties of *Cryptococcus neoformans*, var. *grubii*, also called serotype A, and var. *neoformans*, also called serotype D (Table 1). It was proposed that these varieties be given different species names [10], but a large international group more recently proposed using the term “*Cryptococcus neoformans* species complex” until variations between a large number of strains are more thoroughly analyzed [11]. I will refer to the species complex as simply *C. neoformans* and indicate if particular information is relevant to only one serotype in this review. *Cryptococcus neoformans* has the largest and most metazoan-like haploid genome of the four yeasts, with introns in virtually all genes (typically multiple per gene) and relatively large regional centromeres that all include retrotransposon sequences (Table 1) [12–14]. *Candida albicans* has the next largest haploid reference genome, with several hundred fewer open reading frames (ORFs), very few introns, and relatively small regional centromeres compared to *C. neoformans* [15–18]. An additional noteworthy aspect of that genome is that the CUG codon typically decoded as leucine is decoded as serine in *Candida albicans* [19]. The *Saccharomyces cerevisiae* and *Schizosaccharomyces pombe* reference genomes are the smallest genomes, and despite their similar sizes, *S. cerevisiae* has substantially more ORFs (Table 1) [20–23]. *S. cerevisiae* also has very few introns (similar to *C. albicans*) and small point centromeres, while *S. pombe* has many introns and large regional centromeres (more similar to *C. neoformans*) [21–25].

**Table 1:** Genomic features of reference strains for four model yeasts.^*a*^

Genomic Feature	*Candida albicans*	*Cryptococcus neoformans*^*b*^	*Saccharomyces cerevisiae*	*Schizosaccharomyces pombe*
Haploid size (Mb)	14.3	19.1 (18.9)	12.1	12.6
Chromosome Number	8	14	16	3
ORFs^*c*^	6200	6600 (7000)	6600	5100
Genes with introns	4%	98% (> 99%)	5%	43%
Centromere size (kb)	3–5	20–65	0.125	40–110
Total TE content	0.8%	6.6% (5.9%)^*d*^	3.5%	1.1%
DNA transposons:				
“cut-and-paste”	0.1%	0.7% (0.5%)	0%	0%
*Crypton* or *Helitron*	0%	0.1% (0.1%)	0%	0%
Retrotransposons:				
LTR	0.6%	3.9% (3.1%)	3.5%	1.1%
non-LTR	0.1%	0.5% (0.4%)	0%	0%

^*a*^ See text for references.

^*b*^ Serotype D, serotype A in parentheses.

^*c*^ Including dubious ORFs, rounded to nearest hundred.

^*d*^ Content for this species includes 1.4% (1.9%) unclassified TEs.

### TE content & distribution in the four yeasts

#### Candida albicans

*Candida albicans* is a diploid budding yeast commonly found in the human digestive tract that can switch between distinct cell types and undergo a parasexual cycle, rather than a true sexual cycle [26, 27]. It is an opportunistic pathogen that can cause mucosal infections and more rarely systemic infections, the latter of which are more common in immunocompromised people [28, 29]. The parasexual cycle involves mating of diploid cells to form tetraploid cells, followed by random concerted chromosome loss that reduces DNA content to approximately a diploid state [30–33]. Mating is regulated by a mating type locus (*MTL*) that has **a** and α alleles [27], though same-sex mating has been observed in certain contexts [34]. *MTL* also regulates switching between white and more elongated opaque cell types, and opaque cells mate much more readily than white cells (about 1 million-fold better) [35]. *C. albicans* can grow as yeast, pseudohyphae, and hyphae. Environmental cues, such as growth temperatures of 37 °C, neutral pH, or the presence of serum trigger hyphae formation, and hyphal growth is associated with virulence [26].

The *C. albicans* reference genome (strain SC5314) harbors multiple families of DNA transposons and retrotransposons [16]. Sequences annotated as DNA transposons or transposase genes and retrotransposons or solo LTRs account for approximately 0.8% of the reference genome [16, 36], with retrotransposons and solo LTRs making up the bulk of the elements (Table 1). Solo LTRs result from recombination between LTRs at retrotransposon termini. These recombination events delete the internal sequences and one LTR copy from the genome, and this loss of sequences restricts the accumulation of LTR retrotransposons in genomes. DNA transposons include members of the *Mariner*/Tc1 (Cirt elements) and MULE superfamilies [37–39], but these elements have not been characterized in detail. Thirty-four LTR retrotransposon families and three non-LTR retrotransposon families were identified in early studies of individual elements or surveys of earlier assemblies of the SC5314 reference genome [37, 40–43]. All 34 LTR retrotransposon families and two of the three non-LTR retrotransposon families are represented in Assembly 22 of the reference genome [16], but in fewer copies than reported in the original studies [37, 43]. An analysis of an early draft of the reference genome reported 16 LTR retrotransposon families, Tca1–16, with internal sequences (sequences between two LTRs) that include multiple members of the Ty1/*copia* (Pseudoviridae) and the Ty3/*gypsy* (Metaviridae) superfamilies [37]. However, only nine LTR retrotransposon families are annotated as having internal sequences in the current assembly (Assembly 22) of the reference genome [16].

**Fig. 2:**
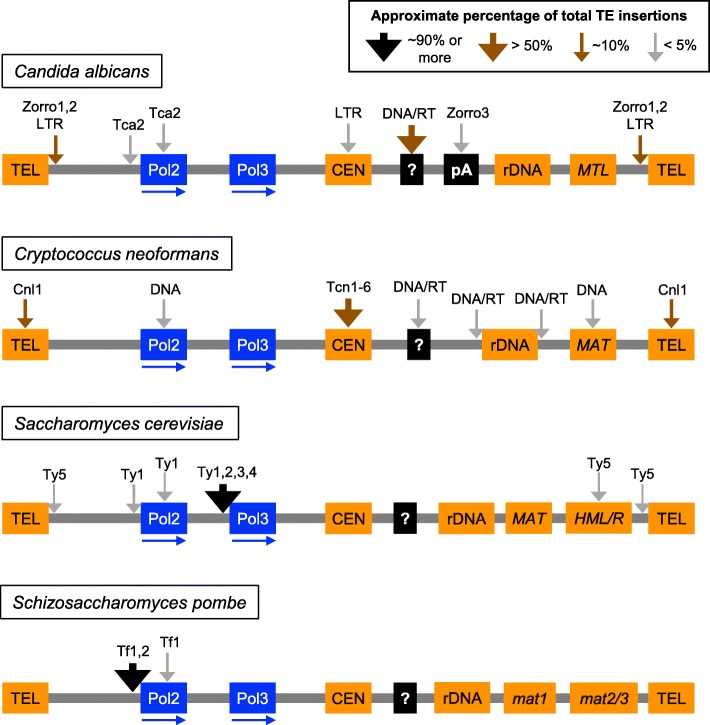
Distributions of TEs in four model yeasts. Gray lines and filled boxes are schematic representations of major DNA sequence features present on chromosomes without any intention of indicating relative size, position, or presence of specific sequences on the same chromosome. Sequence features include: pA- poly(A) sites; CEN- centromeres; HML/R or mat2/3- hidden/silent mating loci; MAT, mat1, or MTL- mating locus; Pol2 or Pol3- RNA Polymerase II or III-transcribed genes; rDNA- ribosomal DNA; TEL- telomeres; ? - unknown/no bias for sequence features. Vertical arrows indicate sites of TE insertions and the relative proportions of total insertions as shown in the key. Names above arrows indicate specific TEs or groups of TEs: DNA- DNA transposons, DNA/RT- DNA transposons or retrotransposons, LTR- LTR retrotransposons or solo LTRs. Blue horizontal arrows indicate the direction of gene transcription.

Potential genomic insertion/distribution biases have not been characterized for *C. albicans* DNA transposons, but some biases have been identified for retrotransposons (Fig. 2). The Zorro1 and Zorro2 non-LTR retrotransposons and some families of LTRs are present in subtelomeric regions [37, 43]. The beta LTRs of Tca8 elements are biased for sequences upstream of tRNA genes [37, 44]. *C. albicans* centromeric DNA is approximately 3–4.5 kb and no shared or common repeat sequences have been identified [17, 18]. Approximately half of centromeres were observed to have one or two LTRs in a pericentric region or within the centromeric DNA [18]. Zorro3 non-LTR retrotransposons are present at sites of poly(A) sequences without any known preference for specific chromosomal regions [43], and newly integrated copies identified by a retrotransposition assay are also targeted to poly(A) sites [45]. Use of a retrotransposition assay also revealed that the Tca2 LTR retrotransposon can insert into ORFs, but prefers to insert in a 300 bp window upstream of start codons [46].

#### Cryptococcus neoformans

*C. neoformans* is a encapsulated budding yeast found in soil, particularly soil contaminated with pigeon guano, that can cause infections in humans by inhalation of spores or cells [47]. Cells can then spread from the lungs to cause infection elsewhere, most commonly cryptococcal meningoencephalitis, and this is particularly likely in immunocompromised people [47]. *C. neoformans* has one mating type locus, *MAT*, with both **a** and α alleles (a heterothallic or self-sterile yeast), but the great majority of clinical and environmental isolates are haploid α cells [48]. Mating between **a** and α cells causes a switch to dikaryotic filamentous growth that can then lead to diploid cells, meiosis, and spore formation [47]. However, same-sex mating between serotype D α cells can produce diploid cells that undergo monokaryotic fruiting, including meiosis and spore formation [49], and diploid α/α cells of serotype A are occasionally observed in environmental and clinical isolates [50]. In addition, cells can undergo phenotypic switching events that change characteristics such as melanin production and capsule size that relate to virulence [47, 51].

The haploid genomes of serotypes A (strain H99) and D (strain JEC21) have multiple families of DNA transposons and retrotransposons [12, 13]. TEs were found to comprise about 5% of the serotype D genome in the original genome sequence report [12]. A more recent study identified 5.9 and 6.6% of the serotype A and D genomes as TEs [52], which is several-fold more than the TE content of the *Candida albicans* reference strain. DNA transposons represent 0.5 and 0.8%, and retrotransposons represent 3.4 and 4.5% of the serotype A and D genomes, respectively (a portion of TEs in each genome was unclassified, Table 1) [52]. Members of the *Crypton*, *EnSpm*, *Harbinger*, *Helitron*, *Mariner/*Tc1, and MULE superfamilies are present, though Helitrons are only present in strain H99 (serotype A) [52]. Cryptons were originally identified in *C. neoformans* as mobile elements consisting of tyrosine recombinase ORFs that lacked any flanking long terminal or terminal inverted repeats – they were flanked only by a 4 bp repeat that appeared to represent a duplication of the insertion site [53].

Retrotransposons in *C. neoformans* have been analyzed in more detail. Both the serotype A and D genomes contain CRE-type non-LTR retrotransposons and multiple families of LTR retrotransposons [52, 54]. LTR retrotransposons, particularly members of the Ty3/*gypsy* superfamily, account for more than half of the total TE content (Table 1) [52]. An early study of the JEC21 genome identified 10 families of LTR retrotransposons (Tcn1–10), five families of solo LTRs, 10 families of retrotransposon fragments, and the Cnl1 CRE-type non-LTR retrotransposon [54]. Members of the 10 LTR retrotransposon and 10 retrotransposon fragment families are evenly split between the Ty1/*copia* and Ty3/*gypsy* superfamilies. Full LTR retrotransposon sequences were only reported for Tcn1–6, of which Tcn6 is the only member of the Ty1/*copia* group [54]. Tcn1 was proposed to use a self-priming mechanism for reverse transcription similar to Tf1/sushi retrotransposons (Tf1 is discussed later for *Schizosaccharomyces pombe*), and Tcn2–5 were proposed to potentially have novel mechanisms of priming [54].

Distribution biases have been noted for many *C. neoformans* TEs (Fig. 2), but no studies have characterized insertion preferences from datasets of newly integrated TEs. Disruption of the *FRR1* gene (encoding the FKBP12 protein) provides resistance to the drug FK506, and selection for FK506 resistance has been used as a transposon trap assay demonstrating that MULE (T1, T2, and T3) and *Harbinger* DNA transposons can insert into ORFs [55–57]. TEs are also enriched at centromeres, at the *MAT* locus, flanking rDNA, and the non-LTR retrotransposon Cnl1 is present in telomeres [12, 54]. *C. neoformans* centromeres span approximately 20–65 kb and contain multiple copies of Tcn1–6 retrotransposons [12–14]. Over 95% of Tcn1–6 copies are present at centromeres [14], and the presence of multiple retrotransposons distinguishes *C. neoformans* centromeres from the other three yeasts (Fig. 2), though a different *Schizosaccharomyces* species does have centromeric retrotransposons [58].

#### Saccharomyces cerevisiae

*S. cerevisiae* has been used for baking and brewing for thousands of years, and many decades ago was developed into an excellent model research organism particularly for biochemistry, cell biology, and genetics. Strikingly, relatively little is known about the ecology and lifecycle of *S. cerevisiae* in the wild, in part due to the focus on practical, commercial, and laboratory research uses of this yeast [59]. While not generally considered a pathogen, there have been rare reports of *S. cerevisiae* infections in humans [60]. *S. cerevisiae* has a *MAT* locus with **a** and α alleles, and can stably grow as haploid **a** or α cells or diploid **a**/α cells [61]. *S. cerevisiae* is a homothallic yeast (self-fertile), because haploid cells can switch mating type by expressing the HO endonuclease to cleave a specific site at the *MAT* locus and repairing the double-strand break using one of two hidden mating loci, *HML* (α) or *HMR* (**a**) [61]. These hidden mating loci are silent copies of the **a** or α mating-type alleles present at distinct genomic sites from *MAT* that can be used to change the allele present and expressed at the *MAT* locus. Many laboratory strains have mutations in the HO gene that prevent switching from occurring. Mating-type switching in a clonal population can create haploid cells of both mating types that readily mate with each other (without nutritional cues) to form diploid cells with two copies of the same haploid genome. Natural populations tend to consist of diploid cells [62], and can be found on tree bark (particularly oak trees), various fruits, and notably have also been found in habitats far from human activity [59, 63, 64].

The only TEs in the *S. cerevisiae* haploid reference genome (strain S288c) are LTR retrotransposons [20, 22]. An initial analysis identified 3.1% of the genome as LTR retrotransposons and solo LTRs, reporting 331 total insertions consisting of 280 solo LTRs or LTR fragments and 51 retrotransposons [65]. More recent analyses reported an overall retrotransposon content of 3.4% or 3.5% [66, 67]. The annotation of the current version of the reference genome includes 383 total LTRs and 50 retrotransposons [22], though 51 full-length retrotransposons have been previously reported [65, 67] as a result of identifying 32 full-length Ty1 elements, as opposed to 31 in the annotated genome. These retrotransposons include five families, Ty1-Ty5, with Ty3 being the only *gypsy*-type (Metaviridae) and the other four *copia*-type (Pseudoviridae) retrotransposons [65]. The relative abundance of these elements in the reference genome is (including solo LTRs): Ty1 > Ty2 > Ty3 > Ty4 > Ty5 [22, 65]. In particular, Ty1 and Ty2 account for 32 (31) and 13 of the 51 (50) full retrotransposon sequences, and Ty1 LTRs account for over half of all LTRs [22, 65, 67]. Ty1 sequences are separated into three subfamilies: Ty1, Ty1′ with divergent *gag* ORF sequences, and Ty1/2 hybrid elements [65, 67]. Ty2 and a subfamily of Ty3 LTRs referred to as Ty3p appear to have been horizontally transferred to *S. cerevisiae* from *S. mikatae* and *S. paradoxus*, respectively [67].

The genomic distribution of all five families is very biased. About 90% of Ty1-Ty4 elements are found in a 750 bp region upstream of genes transcribed by RNA Polymerase III, and all but three such insertions are upstream of tRNA genes, while Ty5 is found at sites of repressive chromatin, including subtelomeres and the hidden mating loci (Fig. 2) [65, 68]. Newly integrated copies of Ty1 preferentially target an approximately 1 kb region upstream of tRNAs and other genes transcribed by RNA Polymerase III [69–72]. About 90% of Ty1 insertions occur within 2 kb upstream of RNA Polymerase III-transcribed genes, ≤ 5% of insertions occur in ORFs, and a similarly low percentage of insertions occur in promoters or other flanking regions of RNA Polymerase II-transcribed genes in both haploid and diploid cells, based on analyses of thousands to over a million sequencing reads for new insertions (Fig. 2) [71, 72]. Nearly all newly integrated copies of Ty3 also insert upstream of RNA Polymerase III-transcribed genes, with the great majority occurring 0–20 bp upstream of the 5′ ends of tRNA genes in the vicinity of the transcription start site [73–76]. There are no functional copies of Ty5 in the reference *S. cerevisiae* genome, but a functional Ty5 element from *S. paradoxus* has been shown to preferentially target heterochromatin in subtelomeric regions and near the *HML* and *HMR* silent mating loci (Fig. 2) [77, 78]. About 76% of newly integrated Ty5 elements target sites of heterochromatin, defined as the region from chromosome ends to 10 kb centromere-proximal of subtelomeric X repeats or *HML* and *HMR* [79]. Integration frequently occurs in intergenic regions upstream of ORFs, peaking around 100 bp and falling to background levels by 1000 bp upstream [79].

#### Schizosaccharomyces pombe

*S. pombe* is a nonpathogenic fission yeast found throughout the world that was developed into an excellent model organism for basic cell biology and genetics research many decades ago [80]. The ecology of *S. pombe* is not well studied, but strains have been isolated from a variety of sources, including cocoa pulp, coffee fruits, grapes, molasses, as well as fermentations of tea (kombucha), sugar cane (cachaca), sorghum (baijiu), and palm sap [81]. The genome has a mating locus, *mat1*, with plus (P) and minus (M) alleles, and two silent mating loci, *mat2-P* and *mat3-M* [61]. Wild-type *S. pombe* strains are homothallic, and can switch mating type by production of a protected single-strand break at *mat1* that results in a double-strand break in one of two daughter cells after another round of DNA replication [80, 82]. This break can be repaired using *mat2-P* or *mat-3 M* [82]. However, mutations can give rise to heterothallic strains, *h+* and *h-*, such as in the 972 *h-* strain used for the reference genome [21, 61]. Strains from natural sources are typically haploid [62], and haploid cells grow stably until they encounter nutrient starvation, which induces mating [80]. Diploid cells typically undergo meiosis soon after they form, unless they are transferred to nutrient-rich conditions [80].

Similar to *Saccharomyces cerevisiae*, the only TEs in the *S. pombe* reference genome (strain 972 *h-*) are LTR retrotransposons [21, 83]. LTR retrotransposons comprise 1.1% of the reference genome [23, 83]. There are 249 LTRs or LTR fragments, 13 full-length retrotransposons, and five retrotransposon fragments containing only portions of internal sequences and sometimes parts of LTRs [83, 84]. Eight of nine families of LTR-retrotransposons are represented only by LTRs or LTR fragments, all full-length retrotransposons are members of the Tf2 family, and the five fragments are Tf1 or Tf2 sequences [83]. *S. pombe* Tf1 elements have been most intensively studied in terms of their replication cycle and integration biases, and full-length Tf1 elements were identified in other wild-type *S. pombe* strains [85]. Tf1 is represented by 28 solo LTRs in the reference genome, compared to 35 solo LTRs for Tf2 [83]. Tf1 and Tf2 have similar sequences and are both members of the Ty3/*gypsy* superfamily of LTR retrotransposons [84, 85]. However, Tf1 and Tf2 are both members of a distinct group of elements that uses self-priming to initiate reverse transcription [86]. There is also a family of 25 ORFs flanked on one or both sides by Tf1 or Tf2 LTRs [21, 83]. There is no evidence that these *wtf* elements are mobile DNA elements, and the ~ 1 kb ORFs have multiple predicted introns, are predicted to encode membrane proteins, and are transcriptionally induced during meiosis [21, 87]. Recent work shows that at least some members of the *wtf* gene family encode poison and antidote proteins that kill spores lacking the particular *wtf* gene, while protecting spores with the gene [88, 89]. These poison-antidote systems allow these genes to act in a selfish manner to increase their transmission to future generations, contributing to the hybrid sterility and reproductive isolation observed for many *S. pombe* isolates [88, 89].

All Tf1 and Tf2 insertions (solo LTRs and full retrotransposons) in the reference genome are present in intergenic regions [83]. They show a strong bias for promoters of genes transcribed by RNA polymerase II, with 83% of insertions closer to the 5′ end of an ORF than the 3′ end, and the bulk of insertions clustered within 400 bp of a start codon (Fig. 2). In contrast to Tf1, new insertions of Tf2 are primarily incorporated into the genome through homologous recombination between Tf2 cDNA and pre-existing Tf2 sequences [90], and large-scale analyses of the minority of Tf2 integration events at nonhomologous sites have not been reported. Nearly all newly inserted copies of Tf1 integrate into intergenic regions, with a strong bias for a 100–400 bp window upstream of RNA Polymerase II-transcribed genes [91, 92]. More recent analyses of tens of thousands or approximately 10 million new insertions show that > 95% of Tf1 insertions are targeted to intergenic regions, particularly within 500 bp of Pol II promoters, and only ~ 3–4% of insertions occur in ORFs (Fig. 2) [93, 94]. Similar distributions are observed in haploid and diploid cells [93], 93% of insertions occur at intergenic sites between tandemly or divergently transcribed ORFs [93], and 80% of insertions are closer to the 5′ end of an ORF than the 3′ end [94].

In summary, these four yeasts have diverse lifestyles, sexual cycles, and genomic features that could result in different constraints and opportunities for the evolution of TEs in these species. Comparative studies of TEs based on these differences could provide important insights into TE biology. For instance, *Candida albicans* has a parasexual cycle that is distinct from the sexual cycles of the other three yeasts. Characterizing TE regulation during the *C. albicans* parasexual cycle in comparison to TE regulation during sexual cycles in the other yeasts could distinguish whether distinct reproductive cycles are characterized by very distinct mechanisms of regulating TEs. The *C. albicans* parasexual cycle also has the potential to generate high levels of genetic diversity, which could include dramatic changes in TE content and diversity. TE content and distribution changes during experimental evolution of *C. albicans* through many rounds of the parasexual cycle could be compared to TE content and distribution in the other three species after evolution through many rounds of their sexual cycles. Such studies could test whether TE content and distribution changes much more rapidly in *C. albicans* or if TEs in this species have any special relationships with their host to protect them from dramatic copy number changes during parasexual reproduction.

The types of TEs present and their abundances also vary between the four species. The *Cryptococcus neoformans* genome has the greatest TE content and diversity, *Candida albicans* has moderate TE diversity, but low TE content, while *Saccharomyces cerevisiae* and *Schizosaccharomyces pombe* have very low TE diversity. Comparative studies could explore whether changes in TE content and diversity have similar impacts on different species. For instance, *S. cerevisiae* strains have been engineered that have up to 10 times the normal content of Ty1 retrotransposons, increasing the total DNA content of the strains by up to 15% [95]. These strains are hypersensitive to DNA-damaging agents, but they exhibit remarkably few other phenotypes, including only modest changes in growth rates [95]. Whether the ability of *S. cerevisiae* cells to tolerate such a large change in TE content depends on specific characteristics of this species or specific aspects of Ty1 regulation and targeting is unknown. Carrying out similar experiments in other species with different types of TEs could address these issues.

Distributions of TEs are also dramatically different in each species (Fig. 2). These distributions reflect both insertion preferences of the TEs themselves and selection processes that act on insertions after integration [96]. TEs are frequently targeted to particular genomic regions through interactions with specific host proteins that have roles in transcription and chromatin regulation, and the three-dimensional arrangement of DNA sequences in the nucleus can also influence targeting [96]. TE distributions in these yeasts likely reflect the evolution of specific TE-host relationships that could impact cellular phenotypes and adaptability [96], and these relationships could be further explored through comparative studies. Expressing TEs from one species in a different species could help address the contribution of TEs themselves, interactions between TEs and conserved proteins or chromatin features, and specialized TE-host interactions to TE distribution biases. For instance, the introduction of the *Candida albicans* non-LTR retrotransposon Zorro3 into *Saccharomyces cerevisiae* resulted in targeted retrotransposition with subtle differences in the outcome of reverse transcription between hosts [97]. This indicates that Zorro3 targeting likely depends on element-encoded proteins or interactions with conserved features of DNA/chromatin.

Expressing TEs from one species in different species could also address other aspects of TE biology. Growing cells engineered to harbor types of TEs not normally present in their genomes for long time periods could provide a means to characterize TE copy number and distribution changes prior to the evolution of specific host-TE interactions. Alternatively, specific TE-host relationships identified in one species could be engineered in a diverse species to further explore relevant mechanisms. TE regulation/activity in the pathogenic yeasts would need to be better characterized for many such studies, though.

Informative comparative studies could also involve analyzing multiple wild strains of one species, or comparing any of the four species to more closely related species. Substantial TE content variation in geographically diverse isolates of *S. cerevisiae* (from a few-fold to approximately 10-fold) provides an opportunity to identify alleles in these strains or changes in the TE-host relationship that may have contributed to these differences, in addition to providing diverse TE landscapes for functional studies [66, 98]. Interestingly, the reference *S. cerevisiae* genome has a high TE content (3.5%) compared with genomes of many other isolates that have ~ 1–2% TE content [66]. The identification of horizontal transfer of Ty2 from *S. mikatae* to *S. cerevisiae* provides the opportunity to compare TE regulation of the same element in these two different host species [67]. *Schizosaccharomyces japonicus* harbors 10 retrotransposon families present near centromeres and telomeres, while three other *Schizosaccharomyces* species (including *S. pombe*) harbor zero to two retrotransposon families and lack retrotransposon sequences near centromeres and telomeres [58]. The reduced presence of retrotransposons in these latter three species is correlated with the evolution of the Cbp1/Abp1 protein family in these species after they diverged from *S. japonicus*, and this protein family regulates centromeric heterochromatin and retrotransposon silencing [58]. The Tj1 *S. japonicus* retrotransposon frequently integrates upstream of tRNA genes at centromeres when expressed in *S. pombe*, consistent with its location at centromeres in *S. japonicus*, but in contrast to *S. pombe* Tf1 and Tf2 [99]. This indicates that Tj1 targets these sites based on conserved chromosomal features or protein interactions. These last two studies provide a good example of how comparative approaches that are followed through to functional assays can begin to provide valuable information about host-TE relationships. In the next section, I highlight differences in the presence and nature of RNA interference in the four yeasts that could contribute to the diversity of their TE landscapes.

### Diversity in RNA interference pathways

There is a wide diversity in the presence and nature of homology-dependent silencing mechanisms among fungi [100]. RNA interference (RNAi) is one such mechanism that minimally depends on production of short-interfering RNA molecules (siRNA) by Dicer proteins (Dcr), followed by loading of small RNAs onto Argonaute proteins (Ago) and into RNA-induced silencing complexes (RISC), resulting in targeted cleavage (slicing) of homologous RNA molecules or other forms of gene silencing (inhibition of transcription or translation) [100, 101]. RNAi in fungi also frequently involves an RNA-dependent RNA polymerase (RdRP or Rdp), similar to what is observed in plants [100, 101]. Endogenously produced siRNAs (18–30 nucleotides) frequently target TEs to repress mobility of these elements (though in animals piwi-interacting RNA, or piRNA, pathways frequently contribute to TE repression) [101]. Not all fungi have RNAi pathways, and the relative importance of RNAi for controlling TEs can also vary [100]. Here, I summarize the main aspects of RNAi (or note its absence) for the four yeast species as an example comparison of TE regulation in these species.

#### Candida albicans

One Ago protein (Ago1), a noncanonical Dcr protein (Dcr1) and a Dicer-like protein that does not appear to be catalytically active (Cdl1) are present in *C. albicans*, but no Rdp homolog has been identified [102–104]. Cell extracts or purified Dcr1 have in vitro Dcr-like cleavage activity [103, 104], and small RNAs corresponding to TEs, including the Zorro elements discussed earlier, have been cloned [103]. However, expression of an inverted repeat construct to generate hairpin RNA as a substrate for siRNA production failed to trigger gene silencing, even though cell extracts could produce small RNAs from the hairpin RNA in vitro [105]. It has been reported that introduction of a synthetic siRNA silenced the *EFG1* gene, but the mechanism of the silencing and the requirement for RNAi proteins was not investigated [106]. Regulation of the relatively low level of TEs in the *C. albicans* genome through RNAi has not been reported.

In contrast, homozygous *dcr1∆*/*dcr1∆* mutants are defective for processing rRNA and snRNA transcripts in *C. albicans*, a function that appears to be independent of Ago1 [104]. Homozygous *dcr1∆*/*dcr1∆* mutants were only recovered when an inducible copy of *DCR1* was integrated into the genome [104]. In the absence of *DCR1* induction, mutants exhibited poor growth and accumulated lower levels of siRNA, but these phenotypes were not seen in *ago1∆*/*ago1∆* mutants [104]. These authors suggest that the role of Dcr1 in rRNA and snRNA processing is responsible for the growth defect of mutants and compare *C. albicans* Dcr1 to the *Saccharomyces cerevisiae* Rnt1 protein, an RNase III enzyme that also contributes to rRNA and snRNA processing.

#### Cryptococcus neoformans

RNAi has been shown to regulate TEs in *C. neoformans* [56, 107]. The *C. neoformans* serotype A genome harbors a single *AGO1* gene, while the serotype D genome harbors two – *AGO1* and *AGO2*, and both genomes have two *DCR* genes (*DCR1*, *DCR2*) and a single *RDP1* gene [12, 102, 107]. Sequences of these RNAi genes are distinctive from homologs in other basidiomycetes, including the absence of a DEAD/DEAH box helicase domain in *DCR1* and *DCR2* [102]. Expression of plasmid copies of inverted repeat constructs designed to produce hairpin RNA to two different genes causes gene silencing, demonstrating the existence of an RNAi pathway in *C. neoformans* [108]. Ago1, Dcr2, and Rdp1 in particular are important for gene silencing by RNAi, and transcriptome analysis shows that multiple DNA transposons and retrotransposons are upregulated in *rdp1∆* mutants [107]. Many small RNAs from mating or vegetative cells correspond to DNA transposons, retrotransposons, and centromeres [56, 109], and centromeres are the major sites of Tcn1–6 LTR retrotransposons [14].

A sex-induced silencing system represses TEs in *C. neoformans* during opposite-sex and same-sex mating, which depends on Ago1, Dcr2, Rdp1, and to a lesser extent Dcr1 [56, 57]. Expression of all four of these proteins is increased at the translational level during mating. Substantial increases in RNA for a *Harbinger* DNA transposon and the Tcn1, Tcn3, and Tcn4 retrotransposons are observed in cells placed under mating conditions for *ago1∆* and *rdp1∆* single mutants, and *dcr1∆dcr2∆* double mutants [56]. RNA levels for T2 and T3 DNA transposons are also increased in *rdp1∆* mutants under mating conditions [57]. Tcn1 H3K9 methylation is reduced in wild type and *rdp1∆* cells during mating, indicating that chromatin changes may lead to increased transcription of retrotransposons during mating [56]. Use of the *FRR1* gene as a transposon trap shows evidence for increased frequencies of gene disruption by DNA transposons during both opposite-sex and same-sex mating [56, 57]. Overall, RNAi protects the *C. neoformans* genome from TE insertions during mating.

While Ago1, Dcr1, and Dcr2 were reported to localize with P-bodies in the cytoplasm, Rdp1 localizes to nuclei [56]. A subsequent study identified a nuclear Rdp1 and Ago1 complex that associates with the spliceosome, the Spliceosomal-Coupled and Nuclear RNAi (SCANR) complex [109]. This complex produces siRNA from transcripts that spend a long time associated with the spliceosome due to inefficiently spliced introns. A number of the transcripts most strongly regulated by this pathway include transcripts for DNA transposase genes [109]. These results indicate that *C. neoformans* has an additional RNAi pathway that represses intron-containing DNA transposons.

#### Saccharomyces cerevisiae

There is no RNAi pathway in *S. cerevisiae* and there are no *AGO*, *DCR*, or *RDP* homologs present in the genome [102, 103]. This loss of RNAi in *S. cerevisiae* and related yeasts has been proposed to have resulted from the presence of double-stranded RNA (dsRNA) killer viruses in these yeasts [110]. In *S. cerevisiae*, cytoplasmic dsRNA M viruses and L-A viruses together result in stable maintenance of killer toxin-producing and toxin-immunity phenotypes [111]. Yeast cells that produce killer toxins and are themselves immune to the toxin outcompete sensitive yeast cells, providing a selective advantage [111]. RNAi is incompatible with maintenance of the dsRNA killer virus system [110], so the presence of an RNAi pathway can create a disadvantage for cells. An RNAi pathway capable of gene silencing can be engineered in *S. cerevisiae* cells by expressing *AGO1* and *DCR1* from the related yeast *S. castellii*. Interestingly, the engineered *S. cerevisiae* cells produce small RNAs that match Ty LTR retrotransposons and repress Ty1 mobility (only Ty1 was tested) [103, 110].

#### Schizosaccharomyces pombe

RNAi has an important role in regulating heterochromatin formation in *S. pombe* [112], but also contributes to silencing of retrotransposons [113]. *S. pombe* has one homolog for each type of RNAi protein – Ago1, Dcr1, and Rdp1 [21, 114]. RNAi proteins repress *dg* and *dh* centromeric repeats that produce sense and antisense transcripts by degrading their transcripts and promoting H3K9 methylation at those sites, a histone mark associated with heterochromatin [112]. A substantial proportion of siRNA corresponds to *dg* and *dh* centromeric sequences, and formation of double-stranded hairpin regions in transcripts from these elements appears to contribute to siRNA formation [21, 115, 116]. Single RNAi component mutants are compromised for centromere function, exhibiting lagging chromosomes during anaphase and reduced cohesin binding at centromeres [117, 118].

In contrast to their significant effects on centromeric repeats, RNAi proteins have only a modest effect on Tf2 LTR retrotransposon expression. Tf2 RNA is reduced only 1.5 to 2-fold in *ago1∆*, *dcr1∆*, and *rdp1∆* single mutants [119]. Dcr1 and Rdp1 bind to LTRs, based on an assay that detects transient binding to chromosomal sites, and LTR RNA levels are increased 6 to 8-fold in single-gene RNAi mutants [120]. However, Tf2 ORF RNA levels are increased by 2-fold or less, again supporting a modest influence on retrotransposon expression [120]. Multiple studies did not detect retrotransposon-derived siRNAs or detected very low levels of retrotransposon-derived siRNAs by Northern blotting and RNA sequencing [115, 116, 120].

A more recent study demonstrated that very low levels of retrotransposon-derived siRNAs are due to exosome-mediated degradation of Tf2 transcripts [113]. Small RNA sequencing in *rrp6∆* mutants compromised for exosome-mediated RNA degradation identified siRNAs corresponding to a number of genomic loci including Tf2 [113]. Ago1 and Dcr1-dependent H3K9 methylation of Tf2 ORFs occurs in *rrp6∆* mutants, and siRNA production depends on Ago1, Dcr1, and Rdp1. Furthermore, Tf2 siRNA production and H3K9 methylation in *rrp6∆* mutants require the Clr4 H3K9 methyltransferase, the Red1 RNA surveillance protein, the poly(A) polymerase Pla1, and the poly(A)-binding protein Pab2. Tf2 RNA is much more abundant when the *rrp6∆* mutation is combined with *ago1∆*, *dcr1∆*, *rdp1∆*, *clr4∆*, or *pla1* mutations [113]. Ago1-dependent Tf2 siRNAs and H3K9 methylation are also observed in wild type cells grown in low glucose [113]. Intriguingly, Tf2 siRNA and H3K9 methylation in *rrp6∆* mutants depends on Nrl1-dependent splicing of a cryptic intron in Tf2 elements [121]. This is reminiscent of the SCANR pathway in *Cryptococcus neoformans* that targets inefficiently spliced RNAs for RNAi [109].

Diversity in the presence and nature of RNAi in these species could form the basis for comparative studies of TE regulation. Only two of the four yeast species regulate TEs through RNAi. In *S. pombe*, RNAi is redundant with other TE restriction mechanisms but also plays an important role in establishing centromeric heterochromatin. In *C. neoformans*, RNAi appears to be a major pathway regulating TEs, and this yeast has the greatest TE content and diversity of the four species. Comparative studies could determine whether species lacking RNAi have a greater diversity of host factors that regulate TEs to make up for the absence of RNAi. Studies could also test whether TEs in species with and without RNAi have evolved to have different relative levels of activity or types of impacts on their hosts.

### Retrotransposition and its regulation in yeasts

**Fig. 3:**
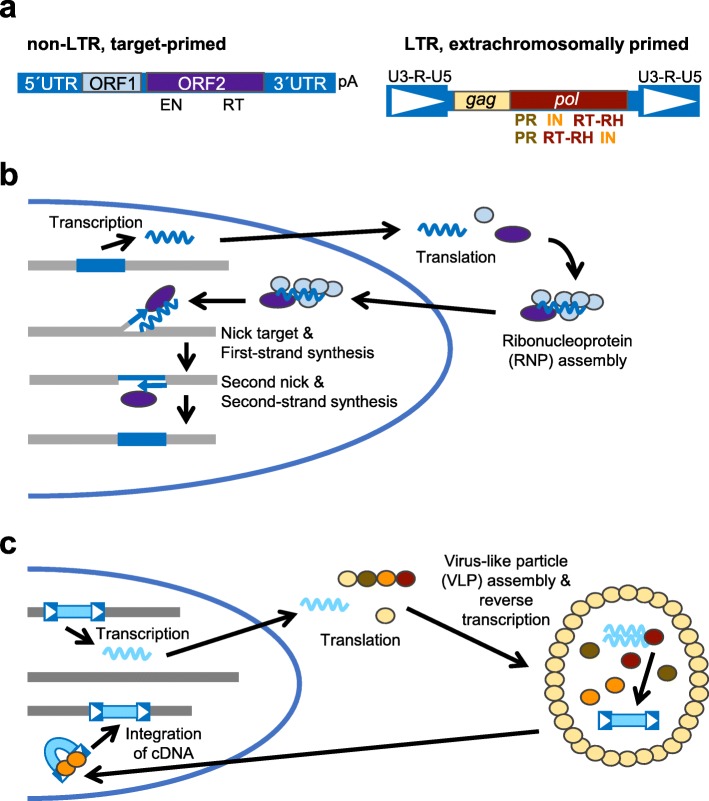
Structure and replication of retrotransposons. **a** Schematic representations of long terminal repeat (LTR) and non-LTR retrotransposons not drawn to scale. Blue boxes with white arrowheads indicate LTRs, and U3-R-U5 indicate regions of LTRs. EN- endonuclease, IN- integrase, pA- poly(A) sequence, PR- protease, RT- reverse transcriptase, RT-RH- reverse transcriptase/RNase H, UTR- untranslated region. **b** Graphic representation of major steps of retrotransposition through target-primed reverse transcription for non-LTR retrotransposons. **c** Graphic representation of major steps of retrotransposition for LTR retrotransposons. Colors of ellipses correspond to proteins from panel **a**, wavy lines represent RNA, boxed arrowheads represent LTRs, and thin blue arrows represent DNA strands newly synthesized by reverse transcriptase.

Retrotransposons are the only TEs in the four yeast species that have been studied in great detail, and LTR retrotransposons are the only type of TEs common to all four genomes. Non-LTR and LTR retrotransposons encode distinct types of proteins and carry out reverse transcription through distinct mechanisms for their mobility (Fig. 3) [122]. Elements in each retrotransposon subclass frequently have two ORFs, though some elements have a single ORF. Transcription of non-LTR and LTR retrotransposons is initiated utilizing internal promoters in 5′ untranslated regions or LTR sequences and terminates at the very 3′ end or in the 3′ LTR, respectively. Translation of non-LTR element ORF1 and ORF2 proteins (or a single protein) is followed by binding of these proteins to the mRNA and movement of the ribonucleoprotein complex to the vicinity of potential chromosomal target sites (Fig.3b). Target-primed reverse transcription takes place when an endonuclease domain (EN) in ORF2p (or in an element’s single ORF protein) nicks a genomic site, providing a DNA end with a 3′-OH that can be extended by the ORF2p reverse transcriptase (RT) using the mRNA as a template. Reverse transcription begins at the poly(A) tail of the mRNA and continues towards the 5′ end of the element, but does not always proceed to the very 5′ end of the mRNA template, which produces 5′ truncated elements. Nicking of the other DNA strand at the target site followed by second strand synthesis results in a new copy of the retrotransposon at the target site, but the details of second strand synthesis have not been characterized (Fig. 3b) [122].

**Fig. 4:**
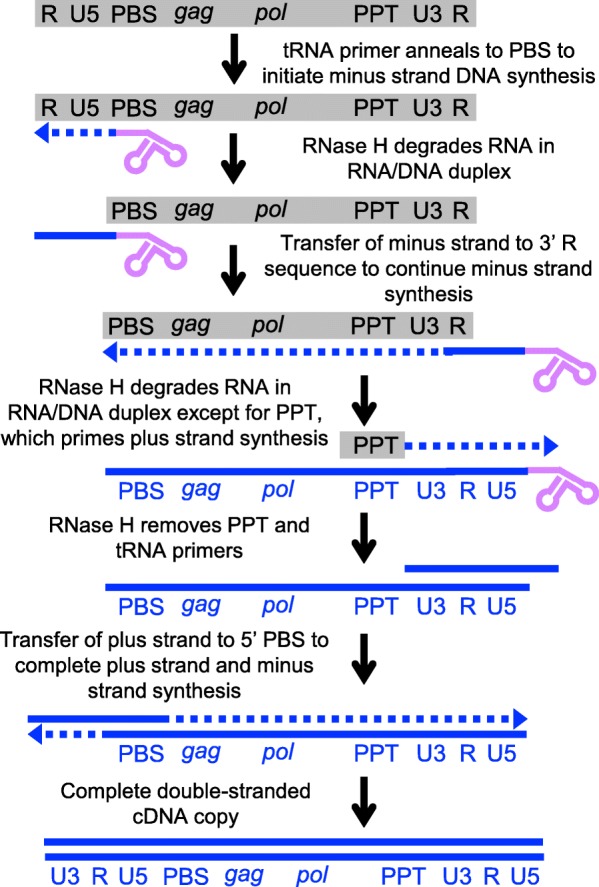
Majors steps of reverse transcription for LTR retrotransposons. The gray box represents retrotransposon RNA with major sequence features not drawn to scale: LTR sequences U3, R, and U5; *gag* and *pol* ORFs; PBS- primer binding site; PPT- polypurine tract. The lavender shape indicates the tRNA primer, dashed blue arrows indicate newly synthesized DNA, and solid blue lines indicate DNA synthesized in a previous step.

Terminally redundant LTR retrotransposon mRNA starts with repeat (R) and unique 5′ (U5) LTR sequences from the 5′ LTR and ends with unique 3′ (U3) and R sequences from the 3′ LTR (Figs. 3a and 4). The mRNA is often translated to produce only Gag protein or less often a Gag-Pol fusion protein by frameshifting between the *gag* and *pol* ORFs (Fig. 3c), though some elements have a stop codon between *gag* and *pol*. Inefficiency of the frameshifting or other mechanisms result in an excess of Gag protein relative to Pol. Gag protein association with the mRNA and Gag-Pol leads to formation of virus-like particles (VLPs) that encapsidate a dimer of RNA and Gag-Pol (Fig. 3c). A protease domain (PR) of Pol cleaves Gag-Pol into Gag, PR, integrase (IN), and reverse transcriptase/RNase H (RT) domains. Reverse transcription occurs in the VLP using a primer binding site (PBS) adjacent to the 5′ LTR that is often complementary to part of a tRNA molecule (extrachromosomal priming, Fig. 4). The initial complementary DNA (cDNA) molecule is a short molecule including U5 and R LTR sequences that can anneal to R sequences at the 3′ end of the mRNA to continue reverse transcription of the minus strand cDNA. RNase H degradation of the mRNA template is incomplete, leaving behind a short purine-rich RNA sequence, the polypurine tract (PPT), that is typically just upstream of the 3′ LTR (Fig. 4). Reverse transcription starting at this point generates a short plus strand cDNA ending in PBS sequences that can anneal to the PBS sequences at the other end of the minus strand cDNA to produce a full plus strand cDNA (Fig. 4 and see [123] for a more detailed discussion of the steps of reverse transcription). IN binds to the double-stranded cDNA to integrate it at a chromosomal site (Fig. 3c) [122].

The mechanism, regulation, and impact of retrotransposition has been much more intensively studied in *Saccharomyces cerevisiae* and *Schizosaccharomyces pombe* than in the other two yeasts. Two retrotransposons (Tca2 and Zorro3) have been studied in some detail in *Candida albicans*, but transposition of particular retrotransposons (or other TEs) in *Cryptococcus neoformans* has not been studied in detail. Further study of TEs in the pathogenic yeast species will facilitate comparative studies of these elements.

#### Candida albicans

**Fig. 5:**
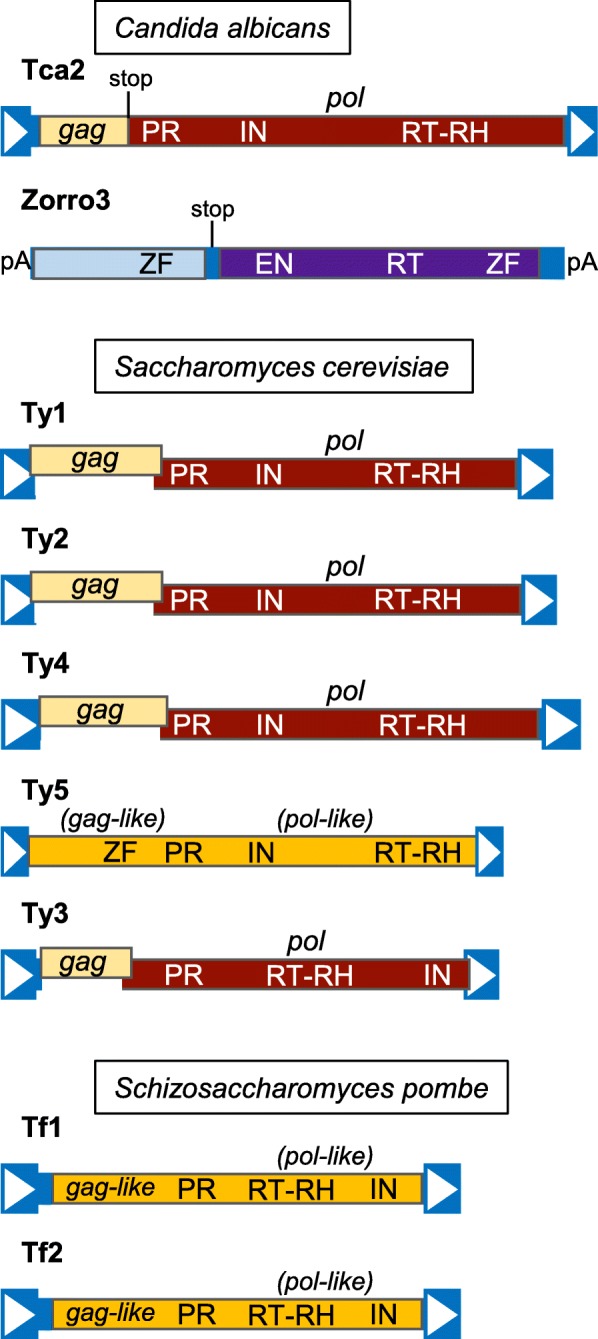
Characterized retrotransposons from three yeast species. Schematic representations of the indicated retrotransposon families drawn to scale. Boxed arrowheads indicate LTRs. Domains of proteins: EN- endonuclease, IN- integrase, PR- protease, RT- reverse transcriptase, RT-RH- reverse transcriptase-RNase H, ZF- zinc finger. pA- poly(A) sequence. The *gag* ORF of Ty1-Ty4 is raised relative to *pol* to indicate a + 1 translational frameshift between *gag* and *pol*. A vertical line with “stop” indicates the site of one or more stop codons.

Retrotransposition of Tca2 LTR retrotransposons and Zorro3 non-LTR retrotransposons has been demonstrated using elements harboring retrotransposition-indicator genes [45, 46]. Tca2 was initially discovered because one particular strain of *C. albicans* accumulates very high levels of extrachromosomal Tca2 DNA [41]. Tca2 is a member of the Pseudoviridae family (Ty1/copia group, IN domain upstream of RT domain in *pol*) flanked by 280 bp LTRs, is 6426 bp long, and has 972 bp and 4728 bp *gag* and *pol* ORFs that are separated by a stop codon (Fig. 5). Tca2 RNA levels are higher at 37 °C than 27 °C, and chromosomal elements harboring a retrotransposition-indicator gene between *pol* and the 3′ UTR retrotranspose at 37 °C [46]. A small set of marked Tca2 insertions shows a preference for integration in a 300 bp window upstream of start codons [46], but the factors responsible for this bias are unknown.

The identification of a stop codon between Tca2 *gag* and *pol* led those authors to suggest that translation of Tca2 Pol protein might rely on stop-codon suppression using an 8-bp purine-rich sequence present immediately after the *gag* stop codon that is followed by a sequence predicted to form a pseudoknot in the RNA [41]. A subsequent study did not find any evidence that a *gag-pol* junction sequence including 150 bp upstream and downstream of the *gag* stop codon could allow read-through translation of a reporter gene [124]. In contrast, deletion of the predicted pseudoknot region in the central portion of the junction sequence increased reporter gene expression, and weak promoter activity in the junction sequence was reported [124]. These authors suggested that the weak promoter activity was unlikely to be responsible for appropriate levels of *pol* expression for retrotransposition, because they found that the promoter was not induced by growth at 37 °C [124], a temperature that induces full-length Tca2 transcription and retrotransposition [46]. The mechanism responsible for expression of appropriate levels of Tca2 *pol* for retrotransposition therefore remains to be determined.

*C. albicans* Zorro3 non-LTR retrotransposons (L1 superfamily) have very short 5′ UTRs and are flanked on both sides by poly(A) sequences (Fig. 5) [43, 45]. Poly(A) sequences are expected at the 3′ end of non-LTR elements due to the mechanism of target-primed reverse transcription, but the presence of poly(A) sequences at both flanks is unusual. Zorro3 is 5763 bp long, with a 1608 bp ORF1 and 3450 bp ORF2 separated by multiple stop codons [43]. Comparison of allelic sites lacking Zorro3 insertions in other strains reveals that Zorro3 integrates into poly(A) sequences [45]. New Zorro3 insertions resulting from a chromosomal Zorro3 element harboring a retrotransposition-indicator gene and expressed from a heterologous promoter are targeted to poly(A) sequences in intergenic regions [45]. Insertions into central regions of poly(A) sequences could explain the presence of poly(A) sequences on either side of Zorro3, and target site duplications could potentially increase the length of these poly(A) tracts. However, the total length of the poly(A) tracts on either side of new Zorro3 insertions can be much more than twice the length of the original poly(A) target [45], indicating that other mechanisms likely contribute to the length of these poly(A) tracts. Many newly inserted copies include the full 5′ end. Zorro3 retrotransposition is temperature-sensitive, with no retrotransposition detected at 37 °C, modest levels at 27 °C, and much higher levels at 22 °C [45].

Zorro3 is also active in *S. cerevisiae*, even though non-LTR elements are not present in that yeast [97]. Expression of a marked Zorro3 element in which all CUG codons were engineered to be UCU (since *C. albicans* CUG specifies serine [19]) results in detectable retrotransposition events. The frequency of new insertions is dramatically reduced by mutations in the zinc finger region of ORF1, as well as the ORF2 EN or RT domains [97]. New insertions are only rarely full-length and show evidence of RT template-switching to other RNA or DNA sequences, in contrast to results in *C. albicans*, but all targeted poly(A) sequences. Comparing Zorro3 insertions in the native host to those in *S. cerevisiae* demonstrates that the Zorro3 integration bias is either independent of cellular factors or relies on factors conserved between *C. albicans* and *S. cerevisiae*.

The distinct temperature preferences of Tca2 and Zorro3 elements are intriguing in the context of *C. albicans* opaque-white cell-type switching. Growth at 37 °C promotes switching to white cells [35] and also promotes Tca2 expression and mobility [46]. Opaque cells are much more mating-competent and are stable at lower temperatures (~ 25 °C) [35], in the range of temperatures permissive for Zorro3 mobility [45]. Also, the Tca1 LTR-retrotransposon is a 5.6 kb nonautonomous element that does not have any ORF sequences, but Tca1 RNA levels are higher at 25 °C than 37 °C [40, 125]. Potential specific connections between retrotransposon regulation, cell-type switching, and mating in *C. albicans* have not been explored.

#### Saccharomyces cerevisiae – overview of retrotransposons

Two analyses of the reference genome identified 51 LTR retrotransposons in *S. cerevisiae*: 32 Ty1, 13 Ty2, two Ty3, three Ty4, and one Ty5 [65, 67]. In addition, two truncated copies each of Ty1 and Ty2, and one truncated copy of Ty4 were identified [67]. The single Ty5 element is not full length, but functional copies of Ty5 from *S. paradoxus* have been studied in *S. cerevisiae* [77, 126], and information presented for Ty5 is based on those elements. Ty3 is the only Metaviridae family member (RT upstream of IN in *pol*), while all other elements belong to the Pseudoviridae family (Fig. 5). LTRs for these elements range from 251 (Ty5) to 371 (Ty4) bp and full-length elements range from 5.4–6.2 kb (Fig. 5) [77, 127–129]. Ty1–4 each have *gag* and *pol* ORFs, with *pol* in the + 1 reading frame, and inefficient frameshifting results in the required excess of Gag to Pol protein for retrotransposition [129–132]. For Ty5, Gag and the functional domains of Pol are processed from a single polypeptide by Ty5 PR, but a Gag to Pol ratio of approximately 5:1 is observed, indicating that the Pol protein domains may be less stable than Gag [126]. Ty1 and Ty3 are the most extensively characterized elements, even though Ty2 is more abundant that Ty3. Ty1 and Ty2 have very similar sequences; relative to Ty1, Ty2 has a 1 bp deletion in its LTR, a divergent *gag* sequence, and a short divergent region in *pol* [133]. Ty1 expression and mobility are substantially higher than Ty2, though, and most studies have focused on Ty1 rather than Ty2 [133].

There are excellent recent reviews that discuss the Ty1 and Ty3 retrotransposition cycles in detail [133, 134], which will not be the focus of the following sections. Instead, I will compare and contrast selected aspects of their regulation with some discussion of Ty5 to consider overall regulation of retrotransposons in *S. cerevisiae*. Multiple large-scale genetic screens for cellular factors (host factors) that regulate Ty1 and Ty3 have identified hundreds of genes that regulate these retrotransposons with roles in cell cycle regulation, chromatin, DNA damage/replication, mRNA degradation/translation regulation, nuclear transport, vesicular trafficking, and other cellular processes [135–141]. Approximately 15% of these genes overlap for Ty1 and Ty3 [134]. The annotation of the reference genome identifies 175 genes that increase retrotransposon activity when mutated and 311 genes that decrease retrotransposon activity when mutated [142]. This large number of regulators likely reflects the complex replication cycle of retrotransposons [133, 134]. It would also be interesting to determine whether the absence of RNAi and related mechanisms in *S. cerevisiae* has resulted in the evolution of a greater number of cellular genes regulating retrotransposons, though, or if the presence of RNAi and similar mechanisms in other organisms masks the role of some cellular factors in TE regulation.

#### Saccharomyces cerevisiae – example host factors regulating Ty1, Ty3, or Ty5

Ty1, Ty3, and Ty5 are all regulated by the pheromone-response pathway (mating-response) in haploid cells, and each is repressed at the transcriptional level by the *MAT*a1/α2 repressor in diploid cells [133, 134, 143]. Ty1 retrotransposition is repressed by treatment with mating pheromone (**a** or α-factor) at a posttranslational step that involves phosphorylation of Ty1 Gag, changes in VLP structure, and reduced cDNA levels [144]. The pheromone-response pathway shares components with the filamentous-growth pathway, which is triggered by poor nutritional conditions and leads to formation of pseudohyphae, and the Fus3 MAP-kinase for the pheromone-response pathway inhibits cross activation of the filamentous-growth pathway [145]. Fus3 activity inhibits Ty1 retrotransposition at transcriptional and posttranslational steps [146, 147]. Increased levels of Gag and Pol proteins associated with VLPs are observed in *fus3∆* mutants, supporting an increase in protein stability or VLP assembly in the absence of Fus3 [146]. The Ste12 transcription factor activates genes in response to both the pheromone-response and filamentous-growth pathways, but Ste12 works together with Tec1 for the filamentous-growth pathway. Ty1 expression and retrotransposition are increased by the filamentous growth pathway and its MAP-kinase Kss1 in both haploid and diploid cells [146, 148], as well as by Ste12 and Tec1 [149–151]. These observations indicate that the filamentous-growth pathway positively regulates Ty1, while the pheromone-response pathway negatively regulates Ty1.

In contrast to Ty1 retrotransposition, both Ty3 and Ty5 retrotransposition are activated in mating cells [143, 152]. Transcription and retrotransposition of Ty5 elements located at typical insertion sites in heterochromatin are substantially increased with exposure of haploid cells to mating pheromone, though only modest effects are observed for an element in euchromatin [143]. Ty3 transcription is strongly induced by exposure to mating pheromone [153, 154]. Ty3 VLPs form and mature in cells arrested in G1 with α-factor, but cDNA is not detected and retrotransposition does not occur until cells are released/recover from arrest [155, 156]. Inhibition of retrotransposition depends on cell cycle arrest itself, rather than other aspects of the mating response [156]. Ty3 retrotransposition frequently occurs in haploid cells of opposite mating types allowed to mate and form diploid cells [152]. Ty3 proteins and RNA form cytoplasmic foci in α-factor-treated cells and mating cells that likely represent sites of VLP assembly [155, 157].

Proteins and mRNA for both Ty1 and Ty3 form cytoplasmic foci, retrosomes, that appear to be precursors to VLP formation and associate with cytoplasmic processing bodies (P-bodies) to differing degrees [155, 157–159]. P-bodies are cytoplasmic protein-RNA granules that can regulate mRNA degradation and translation [160]. Ty3 retrosomes formed in response to mating pheromone or expression from an inducible heterologous promoter frequently localize with or are found very near to foci of P-body factors Dcp2, Dhh1, Lsm1, Pat, and Xrn1 [155, 157], which have roles in either mRNA decapping/degradation or translational repression [160]. Expression of Ty3 from an inducible promoter also increases the size or leads to formation of P-body protein foci [157]. Fewer retrosomes are observed in *dhh1∆*, *lsm1∆*, and to a lesser extent *xrn1∆* and *dcp2∆* mutants [155]. Moreover, *dhh1∆* and *xrn1∆* mutants have reduced Ty3 retrotransposition, and *dhh1∆*, *lsm1∆*, and *xrn1∆* mutants affect RNA levels or packaging in VLPs to reduce cDNA levels [138, 155]. Ty1 cDNA and retrotransposition are also reduced in P-body component mutants, such as *dcp2∆*, *dhh1∆*, *lsm1∆*, *pat1∆*, and *xrn1∆*, without substantial effects on Ty1 RNA and protein levels, though the absence of Xrn1 severely reduces RNA packaging in VLPs [158, 159]. Retrosome formation is also greatly reduced in mutants lacking P-body proteins [158]. Unlike Ty3, though, Ty1 retrosomes show only limited overlap with foci of P-body proteins [159], and retrosomes do not form when P-body formation is triggered by glucose deprivation [158, 161]. Despite regulation by common factors, Ty1 and Ty3 again show distinct interactions with the cellular environment.

The positive role of P-body components in Ty1 and Ty3 retrotransposition is intriguing in comparison to TE regulation in other organisms. For instance, Ago1, Dcr1, and Dcr2 proteins localize to P-bodies in *Cryptococcus neoformans* [56], indicating that P-bodies could be sites of TE repression in this yeast. The situation in *C. neoformans* is more similar to the many other organisms in which Ago and Dcr proteins repress TEs [101], because Ago proteins, siRNAs, and microRNAs can be found at P-bodies in animal cells [162]. Mammalian LINE-1 (L1) retrotransposon protein-mRNA foci only sometimes have been localized to P-bodies but have also been detected in stress granules [163–165], related protein-RNA granules that regulate translation of sequestered RNAs [160]. L1 association with P-bodies can lead to degradation of L1 ribonucleoproteins through autophagy [165]. Studies that contrast mechanisms responsible for the positive influence of P-body factors on TEs in *S. cerevisiae* with potential negative regulatory mechanisms in *Cryptococcus neoformans* would provide a more complete understanding of the roles of P-body factors in TE regulation.

The nuclear envelope does not break down during cell division in yeasts, which means that retrotransposon cDNA and integrase protein (at minimum) must be able to enter the nucleus through nuclear pore complexes (NPC) consisting of nucleoporin proteins (Nups) [166]. Ty1 and Ty3 each have basic bipartite nuclear localization signals in C-terminal regions of their IN proteins that are required for IN nuclear localization and retrotransposition (Ty1) [167, 168] or IN nucleolar localization and in vitro integration (Ty3) [169]. Ty1 IN nuclear localization is known to rely on Ran, the karyopherin alpha protein Srp1, the karyopherin beta protein Kap95, and IN interacts directly with Srp1 [170]. Certain Nup mutants decrease Ty1 retrotransposition [136, 140, 141], while some Nup mutants increase and others decrease Ty3 retrotransposition [137, 138]. A more systematic analysis of Ty1 retrotransposition in 19 NPC mutants identified two Nups that restrict retrotransposition and nine Nups that contribute to retrotransposition [171]. Ty3 Gag in VLPs and recombinant Gag interact with Nups containing GLFG (glycine-leucine-phenylalanine-glycine) repeats, and the nucleocapsid domain of Gag enters the nucleus when expressed on its own in a manner that depends on GLFG proteins [172]. The interaction of Gag with Nups was proposed to lead to structural changes in VLPs at nuclear pores, promoting nuclear entry of cDNA, IN, and possibly other VLP components [172].

The roles of many NPC proteins in Ty1 retrotransposition have been studied in greater detail. As just noted, 11 of 19 Nups tested (of about 30 total NPC proteins [166]) regulate Ty1 retrotransposition frequencies, but different Nups have variable and usually modest effects on Ty1 RNA, Gag, and cDNA levels [171]. Interestingly, none of the Nups is required for the nuclear localization of the Ty1 IN domain when expressed on its own. The common theme identified is that all Nups tested, including those that do not affect retrotransposition of a Ty1 marked with a retrotransposition-indicator gene, contribute to the targeting of Ty1 upstream of tRNA genes [171]. Most Nups are required for high frequencies of insertions upstream of two target tRNA genes, and partial deletions of two nuclear basket Nups (Nup1 and Nup60) affect which sites upstream of tRNA genes are targeted. This is not always simply due to lower Ty1 integration, though, because the absence of Nup1, Nup60, Mlp1, or Mlp2 nuclear basket proteins leads to frequent Ty1 insertions into subtelomeric regions [171].

Another recent study of Ty regulation by nucleoporins provides an excellent model for using a comparative approach to probe the coevolution of retrotransposons and their host cells. Nucleoporins from 29 *Saccharomyces* species were examined for high rates of nonsynonymous substitutions relative to synonymous substitutions as a way to identify proteins that have been recently evolving to control retrotransposons [173]. *NUP84* and *NUP82* were among the Nup genes that appear to be evolving under positive selection. *NUP84* is known to contribute to Ty1 and Ty3 retrotransposition [136, 138, 140], while *NUP82* is an essential gene not previously identified as a Ty regulator, but expression of a dominant-negative Nup82 protein reduces Ty1 retrotransposition [173]. Replacing the *S. cerevisiae NUP84* allele with an allele from one of three other *Saccharomyces* species (*S. mikatae*, *S. kudriavzevii*, or *S. bayanus*) leads to moderate but significant increases or decreases in Ty1 retrotransposition, without affecting Ty3 mobility [173]. Similar experiments for *NUP82* identified two different alleles that specifically increased either Ty1 or Ty3 retrotransposition [173]. These results indicate that the corresponding *S. cerevisiae* alleles have evolved to be more restrictive or permissive for specific Ty elements. Overall, this type of approach provides a more sophisticated perspective on the evolution of retrotransposon regulation.

#### Saccharomyces cerevisiae – Ty1, Ty3, and Ty5 insertion biases & regulation by stress.

The insertion biases of Ty1, Ty3, and Ty5 are all due to specific interactions between their IN proteins and cellular factors present at their preferred target sites, which regulate the mutagenic potential of these elements. Ty5 is the most distinct of the three, with an integration bias for heterochromatin near telomeres and the silent mating type loci [77]. This bias is achieved by an interaction between a targeting domain of six amino acids in Ty5 IN that interacts with the Sir4 heterochromatin protein [174, 175]. This interaction provides the initial localization of IN and cDNA to heterochromatin, but the specific site of insertion then appears to depend on the presence of nucleosome-free regions that are accessible for integration [79]. Ty3 integrates 0–20 bp upstream of tRNA genes, near their transcription start sites [73–76], which is dependent on the transcriptional competence of the RNA Pol III promoter [74, 75]. Recapitulation of targeted insertions in vitro using VLPs, an RNA Pol III target gene, and purified RNA Pol III transcription factors shows that integration depends on the Pol III transcription factors TFIIIB and TFIIIC [176]. Specifically, TFIIIB components TATA-binding protein (TBP) and Brf1 are the minimum factors needed for targeted insertions, though Bdp1 contributes to integration efficiency, and TFIIIC is required only when needed to load TFIIIB at a promoter [177, 178].

Ty1 most often integrates within ~ 1 kb upstream of RNA Pol III genes [69, 70], which depends on the transcriptional competence of the RNA Pol III-gene promoter and shows a periodicity that depends on nucleosome positioning and structure [70–72]. This Ty1 integration bias is due primarily to specific interactions between a C-terminal region of Ty1 IN and the RNA Pol III subunit Rpc40 [179], as well as Rpc53, Rpc34, and Rpc31 [180]. An initial screen that identified the Rpc40-Ty1 IN interaction also identified interactions with Ty2 and Ty4 IN domains, but this was not further investigated [179]. Temperature-sensitive *rpc40* and *rpc34* alleles reduce integration upstream of a tRNA gene and retrotransposition of a Ty1 marked with an indicator gene, without substantially altering overall tRNA gene expression [180]. Replacing the essential Rpc40 protein with the corresponding protein from *Schizosaccharomyces pombe* very strongly reduces integration upstream of RNA Pol III-transcribed genes, without having a strong effect on overall retrotransposition frequencies [179]. Interestingly, expression of the *S. pombe* Rpc40 protein results in frequent mistargeting of Ty1 to telomeres and subtelomeres [179], similar to the effect of certain Nup mutants [171].

A variety of stress conditions can influence Ty element expression and mobility, particularly for Ty1. For example, Ty1 and Ty3 transcription and Ty1 retrotransposition are increased in response to adenine starvation [181, 182]. Ty3 retrotransposition is inhibited by high growth temperature (37 °C) or ethanol stress by a mechanism that blocks VLP formation and results in degradation of Ty3 proteins [183]. Ty1 retrotransposition is very temperature sensitive, with an optimum temperature around 20 °C [184, 185], which is at least partly due to temperature-sensitivity of the Ty1 protease [185]. Ethanol stress increases Ty1 transcription [186], oxidative stress increases Ty1 retrotransposition [187], and nutritional stress increases Ty1 transcription and retrotransposition through the filamentous-growth pathway previously discussed [146, 148]. Both Ty1 and Ty3 are restricted by factors that contribute to DNA repair or replication [133, 134], and Ty1 retrotransposition is also increased by exposure to various DNA-damaging or replication-stress agents, including ionizing radiation, hydroxyurea, methylmethane sulfonate, and 4-nitroquinoline-1-oxide [133]. DNA damage signaling through the Rad24 and Rad9 proteins increases processing of Ty1 Pol proteins, RT activity, and cDNA formation to increase retrotransposition [188].

It is important to highlight a few additional aspects of Ty1, considering that it is the most active and abundant retrotransposon in many *S. cerevisiae* strains. Ty1 RNA is extremely abundant in haploid cells, accounting for approximately 0.1–0.8% of total RNA and 5–10% of poly(A) RNA [189, 190], and has a half-life of several hours [191]. Much of the regulation of Ty1 therefore occurs at the posttranscriptional level. Ty1 is also regulated through copy-number-control (CNC), by which increasing copy numbers of Ty1 result in decreasing levels of retrotransposition [192]. Ty1 produces short antisense RNAs that initiate in the *gag* region, have an inhibitory effect on Ty1 retrotransposition, and were considered as potential mediators of CNC [193, 194]. Antisense RNAs are repressed by the 5′-3′ mRNA decay proteins Dcp1, Dcp2, and Xrn1 [193], consistent with the contribution of these proteins to Ty1 mobility [158, 159]. Antisense RNAs are also repressed by the Tye7 transcription factor when Ty1 is activated during adenine starvation [182]. Antisense RNAs were initially reported to inhibit Ty1 transcription [193], but subsequent work shows that antisense RNAs associate with VLPs, reducing levels of mature Pol proteins and RT activity [194]. More recently, Ty1 CNC was shown to depend on production of a truncated Gag protein produced from an internally initiated transcript [195]. This truncated Gag strongly inhibits Ty1 retrotransposition by interacting with the normal Gag protein to decrease VLP assembly and alter VLP structure [195, 196]. Regulation of Ty1 by some ribosome biogenesis proteins and the Mediator transcriptional co-activator occurs at least in part by altering the expression of this truncated Gag protein [197, 198]. Ty1 self-restriction may have evolved as a means of Ty1 stably remaining in an organism that lacks genome-wide small RNA-based repressive mechanisms to limit potentially deleterious unrestricted retrotransposition.

#### Schizosaccharomyces pombe

Thirteen Tf2 LTR retrotransposons are the only full-length TEs in the reference *S. pombe* genome [83], but full-length active Tf1 elements are present in other wild type strains [85]. Tf1 and Tf2 are members of the Metaviridae (Ty3/gypsy) group, approximately 4.9 kb in length, have 358 or 349 bp LTRs, and have single ORFs encoding proteins of 1330 or 1333 amino acids (Fig. 5) [85, 199]. Their RT and IN regions are extremely similar in sequence, they share similarities in regions of their LTR sequences, but their *gag* regions are divergent [85, 199]. The single ORF of each element is translated into one polypeptide that is proteolytically processed into Gag and Pol domains by the PR activity [90, 200]. While Tf1 processing results in mature Gag (capsid, CA), PR, RT, and IN proteins, as expected, Tf2 processing does not separate PR from RT [90, 201]. Retrotransposition of Tf1 and Tf2 has typically been studied by expression of plasmid copies of the elements from inducible promoters [90, 200, 202]. Tf2 mobility, protein levels, and cDNA are much lower than Tf1, and most Tf2 insertions occur by homologous recombination with pre-existing Tf2 sequences [90]. As a result, studies of retrotransposition in *S. pombe* have focused on Tf1, though many studies of transcriptional regulation have focused on Tf2 elements in the reference genome. An excellent recent review addresses details of the steps of Tf1 retrotransposition and regulation of Tf1 and Tf2 by cellular factors [84], so only certain aspects of Tf1 and Tf2 retrotransposition and regulation will be highlighted in this review.

Two aspects of Tf1 retrotransposition are unusual compared to many other LTR retrotransposons. First, the excess of Gag relative to Pol does not result from differences in translation, but apparently results from increased Pol degradation relative to Gag. Gag and IN (as a measure of Pol) are present in a 26:1 ratio in purified VLPs and stationary phase cell extracts [201]. During log phase there are similar levels of each protein, but as cells continue into stationary phase, IN levels decrease substantially, as cDNA levels increase [201]. These results support degradation of IN, and presumably other Pol proteins, as cells reach stationary phase, but the mechanism is not known. Maturation of VLPs and increased cDNA synthesis as cells approach stationary phase contrasts with Ty1 elements in *Saccharomyces cerevisiae*, because Ty1 retrotransposition decreases as cells approach stationary phase, which is correlated with more unprocessed or posttranslationally modified Gag [203]. Second, Tf1 is the founding member of a group of retrotransposons that initiate reverse transcription through a self-priming mechanism [204], rather than using a tRNA primer. The first 11 bases of the Tf1 RNA are complementary to the PBS, and RNA structures form that allow base pairing of these two regions, followed by RNase H cleavage between bases 11 and 12 to generate a short RNA primer for reverse transcription [86, 204–206].

Many cellular factors regulate retrotransposon RNA levels in *S. pombe* in addition to the RNAi and exosome pathways previously discussed. Tf2 transcription is repressed by many proteins, including the chromodomain protein Swi6, the HIRA histone chaperone complex, the histone methyltransferases Clr4 and Set1, the RSC chromatin remodeling complex subunit Sfh1, the histone deacetylases Clr3, Clr6, and Hst4, as well as the Nts1 protein present in one specific Clr6 complex [115, 119, 120, 207–213]. Furthermore, the CENP-B homologs Cbp1/Abp1, Cbh1, and Cbh2 that are involved in establishing centromeric heterochromatin also negatively regulate Tf2 transcription [208]. CENP-B proteins are homologous to transposases of DNA transposons [214, 215], and regulation of Tf2 by CENP-B proteins has been noted as an example of cells co-opting sequences from one type of TE to regulate a different type of TE [208]. Cbp1/Abp1 reduces insertion of Tf1 cDNA into the genome through homologous recombination, recruits Clr3 and Clr6 to Tf2 sequences, and organizes Tf2 retrotransposon sequences into 1–3 nuclear foci, named Tf bodies [208]. Additional factors, such as Set1, Clr3, Clr6, Hst2, Hst4, and the Ku heterodimer are also required for Tf body formation [211, 216]. Ku however does not repress Tf2 transcription, but Ku binding to Tf2 and Tf body formation are reduced by H3K56 acetylation that occurs during S-phase and in response to DNA damage [216].

Incorporation of Tf2 into Tf bodies represses Tf2 mobility [217]. Loss of Cbp1/Abp1 or Set1 each disrupt Tf body formation and results in high mobility of an endogenous Tf2 marked with a retrotransposition-indicator gene [217]. In contrast, absence of individual subunits of the HIRA complex increases Tf2 transcription, but does not prevent Tf body formation, and results in almost no change in Tf2 mobility [217]. Domains of Set1 have different contributions to Tf2 repression and Tf body formation [218]. Use of mutant Set1 proteins compromised only for repression or for Tf body formation shows that loss of Tf body formation increases Tf2 mobility even when repression is maintained, but loss of repression when Tf body formation is maintained does not increase Tf2 mobility [217]. Overall, these results strongly support a repressive role of Tf body formation that is distinct from transcriptional repression. Tf2 transcription is activated by the Sre1 transcription factor in low oxygen conditions [219], and mobility of the marked endogenous Tf2 also increases in response to constitutive Sre1 activity [217].

These observations together with those regarding RNAi and exosome-mediated repression of Tf2 highlight the many layers of cellular defense against retrotransposons. As a further example, the nuclear poly(A)-binding protein Pab2 moderately represses Tf2 RNA levels, but the absence of either Pab2 or Rrp6 (exosome component) can partly suppress the increase in Tf2 RNA observed in the absence of Cbp1/Abp1 [220]. Double mutants lacking Cbp1/Abp1 and Pab2 or Cbp1/Abp1 and Rrp6 accumulate higher levels of Tf2 antisense RNAs than mutants lacking only one of these proteins, which was proposed to trigger increased RNAi-mediated repression of Tf2 [220]. Tf2 antisense RNA increases during middle stages of meiosis, at which point Tf2 mRNA is low, and decreases at later stages, when Tf2 mRNA is high [220]. Antisense RNA may therefore restrict Tf2 retrotransposition during certain stages of meiosis. The Red1 protein that contributes to degradation of meiotic mRNAs also represses Tf2 [221], indicating either that Red1 could contribute to antisense mediated regulation of Tf2 or that multiple lines of defense restrict Tf2 during meiosis.

In contrast to *S. cerevisiae* Ty1 and Ty3, Tf1 Gag protein exclusively localizes to nuclei in the great majority of stationary phase cells [222], a phase in which retrotransposition is observed [200, 202]. Cosedimentation of Gag, IN, and cDNA supports the interpretation that nuclear Gag signal represents VLPs [222]. Curiously, the Pst1 protein that is present in one specific Clr6 histone deacetylase complex is required for this nuclear localization, with many cells lacking Pst1 showing only cytoplasmic or a mix of cytoplasmic and nuclear Gag [222]. Furthermore, Pst1 positively contributes to Tf1 retrotransposition and cDNA recombination [222], despite the repressive effect of Clr6 noted earlier [119]. The nucleoporin Nup124 interacts with Gag and is also required for Gag nuclear localization [223]. The absence of Nup124 substantially reduces Tf1 retrotransposition without affecting Tf1 protein or cDNA levels [223]. The first ten amino acids of Gag is critical for nuclear import and retrotransposition, but amino acids 20–30 cause nuclear import to specifically depend on the presence of Nup124 [224]. As previously discussed, Ty3 Gag also interacts with nucleoporins, though Ty3 Gag does not localize to the nucleus in the context of VLPs [172], as for Tf1.

Several stress conditions are known to regulate Tf1 expression and retrotransposition, but Tf1 does not show a general response to all stress conditions. Treatments with DNA-damaging agents (bleomycin and 4-nitroquinoline-1-oxide), hydroxyurea, osmotic stress, microtubule depolymerizing agents, and nitrogen starvation do not regulate retrotransposition of Tf1 expressed from an inducible promoter [91]. Retrotransposition frequencies are also not changed over a wide range of growth temperatures (22 °C to 36 °C) [202], which is different from what was discussed for Ty1 and Ty3. Furthermore, Tf1 expression is not activated by Sre1 in response to low oxygen, as Tf2 expression is [219]. Tf1 transcription is induced by a short heat stress (39 °C for 15 min) or a low dose of hydrogen peroxide, but not osmotic stress or cadmium [225]. Retrotransposition of at least some individual elements also increases in reponse to cobalt, zinc, caffeine, or phthalate [226]. There is only limited overlap between the stresses that regulate Tf1 and those that regulate Ty1 in *S. cerevisiae*.

Tf1 shows a strong integration bias for promoters of RNA Pol II-transcribed genes [93, 94] that is determined by interactions between IN and specific DNA-binding proteins. Tf1 IN interacts with the transcriptional activator Atf1, which can direct integration events to promoters containing Atf1 binding sites in plasmid targets [227, 228]. Integration at specific promoter regions does not depend on high levels of transcriptional activity of those promoters, though [227]. The essential Sap1 DNA-binding protein plays a role in mating-type switching and can arrest replication forks [229–231]. A hypomorphic *sap1* allele strongly reduces Tf1 retrotransposition without reducing Tf1 protein or cDNA levels [232]. Approximately 63% or 73% of Tf1 insertions occur at sites bound by Sap1, but not all Sap1-binding sites appear to be targeted [232, 233]. This could reflect a need for a threshold level of Sap1 binding to target integration [232]. However, Sap1 replication-fork barrier function may also be needed for integration, based on a preference for integration to occur on the blocking side of the barrier [233]. DNA structures, proteins, or chromatin marks at stalled replication forks may therefore contribute to Tf1 integration. Sap1 preferentially binds within LTRs and adjacent to retrotransposons, which is consistent with a role for Sap1 in Tf1 targeting [234]. Sap1 and Tf1 IN interact when tested in a yeast two-hybrid system, supporting a role for Sap1 in targeting integration events through direct or indirect IN binding [232, 233]. *Saccharomyces cerevisiae* tRNA genes act as replication fork barriers [235], so there are parallels between integration of Tf1 and Ty1–4.

A large-scale screen for factors that specifically contribute to Tf1 integration identified 61 genes that promote integration [236]. The proteins of these genes contribute to diverse cellular functions, including chromatin structure, DNA repair, nuclear and vesicular transport, splicing and mRNA processing, transcription, and translation. While it is not immediately apparent how proteins involved in some of these activities contribute to integration, Tf1 IN interacts with at least two of these proteins in a two-hybrid system: Cwf3, a subunit of a splicing complex, and the Rhp18 DNA repair protein [236]. Some of the proteins identified are homologs of proteins shown to regulate Ty1 and Ty3, supporting common aspects of retrotransposon regulation in *Schizosaccharomyces pombe* and *Saccharomyces cerevisiae* [236].

### Impact of retrotransposons on yeast genomes

Retrotransposons can have diverse effects at the level of individual genes as well as on chromosome structure that produce genetic and phenotypic variation in their host cells. There are not many examples of specific impacts of most of the TEs in the four yeast species, but many studies illustrate how Ty1 and Tf1 alter their respective host genomes. Both Ty1 and Tf1 can disrupt protein-coding genes on occasion, as seen by screening for mutations in specific target genes for Ty1 [237, 238] and large-scale analyses of each element showing occasional integration into ORFs [71, 72, 93, 94]. These retrotransposon-induced mutations would often be deleterious or neutral, with rare mutations conferring a selective advantage.

Ty1 and Ty3 integration most often occurs upstream of tRNA genes and can have modest positive influences on the expression of neighboring tRNA genes [239, 240]. It is not clear, though, how much of a phenotypic impact those changes could have. Ty1 also occasionally integrates upstream of RNA Poll II-transcribed genes, which can disrupt their promoters or change their pattern of expression based on the presence of Ty1 transcriptional control sequences [129, 133]. The latter outcome more often occurs when Ty1 is in the opposite orientation of the targeted gene. For example, induction of Ty1 by adenine starvation can lead to insertions upstream of RNA Pol II-transcribed genes that cause those genes to be more highly expressed in response to adenine starvation [241].

However, Tf1 integration almost always occurs into promoters of RNA Pol II-transcribed genes [93, 94]. Tf1 insertions in either relative orientation can compensate for disruption of target gene promoters by providing transcriptional control sequences that promote expression of neighboring genes [227]. Tf1 also shows a preference for integrating into promoters of stress-responsive genes [93]. Individual strains harboring newly integrated Tf1 elements frequently exhibit moderately increased expression of neighboring genes, rather than reduced expression [225]. Heat stress (39 °C) increases Tf1 transcription and can further increase expression of neighboring genes or increase expression of genes that were not originally affected by the insertion [225]. It was noted that the specific genes upregulated by Tf1 are themselves regulated by heat stress in the absence of Tf1, indicating that Tf1 integration into stress-responsive promoters could provide a means of amplifying stress-dependent gene expression [225].

Particular Tf1 insertions can also provide selective advantages to cells in specific environmental contexts. A library of > 40,000 strains each harboring a single newly integrated Tf1 grown together for many generations in the presence of a moderate concentration of cobalt (a stress that can activate Tf1) showed that while thousands of strains became underrepresented, a subset of > 100 strains became consistently overrepresented in replicate final populations [226]. These latter strains had Tf1 insertions that were frequently near genes that are induced by cadmium or peroxide, or genes regulated by the TOR pathway involved in nutrient sensing and cell growth. The TOR pathway was shown to provide resistance to cobalt, indicating that changes in expression of TOR-regulated genes due to neighboring Tf1 sequences could account for overrepresentation of those strains [226]. Strains harboring specific individual Tf1 insertions outcompeted wild type strains when grown in cobalt, but showed a growth disadvantage in the absence of the stress [226]. Overall, these observations support a role for Tf1 in adaptive gene expression changes conferring increased resistance to particular stresses.

Ty1 is known to have several other types of impacts on its host genome due to its reverse transcriptase activity and its repetitive nature. Ty1 occasionally reverse transcribes cellular RNAs to produce processed pseudogenes or retrosequences that are incorporated into the genome [242, 243]. In particular, subtelomeric Y′ element RNA is preferentially packaged into VLPs and Y′ is mobilized at high frequencies by Ty1 in telomerase-negative mutants [244]. Besides duplicating genomic sequences, this activity can replace intron-containing genes with intron-lacking cDNA copies and potentially put coding sequences under the control of nearby heterologous promoters. Ty1 cDNA can also be captured at sites of double-stranded breaks during DNA repair, which may facilitate DNA repair [245, 246]. Ty1-dependent retrosequences can be incorporated at sites of chromosomal rearrangements, raising the possibility that their incorporation could also contribute to DNA repair [247].

The presence of many Ty1 sequences throughout the genome provides substrates for non-allelic homologous recombination to repair DNA damage. Chromosomal rearrangements that occur spontaneously or in response to DNA damage frequently have Ty1 sequences at their breakpoint junctions [248–250]. Experimental evolution of cells in nutrient-limited conditions can result in adaptive chromosomal rearrangements that frequently form by recombination between Ty1 sequences [251, 252]. Such rearrangements can delete or amplify genes, for instance, producing adaptive phenotypes in response to various stresses or selective conditions [253, 254]. The presence of Ty1 sequences at intergenic sites prevents gene disruptions during these events, and some events are readily reversible in the absence of selective pressure [254]. The introduction of Ty1 sequences at particular chromosome sites can greatly increase the frequency of chromosomal rearrangements at those sites [255]. The presence of adjacent inverted Ty1 sequences or multiple Ty LTRs in different orientations can promote DNA breaks and create hotspots for chromosomal rearrangements (fragile sites), especially under conditions of replication stress [256, 257]. Ty1 sequences (and Ty2) are genomic sites of R-loops [258], in which one strand of DNA at a chromosomal site is hybridized to RNA, and R-loops cause DNA breaks and replication stress [259]. The most frequent target sites of Ty1, tRNA genes, act as replication fork barriers/pause sites based on formation of transcription initiation complexes at their promoters [235]. The presence of Ty1 elements at these sites could be advantageous by allowing for recombination-mediated restart/repair of stalled/collapsed forks [133].

The frequent involvement of Ty1 in chromosomal rearrangements could simply reflect the abundance of Ty1 sequences in the genome, or it could reflect an active role for Ty1 in these processes. DNA-damage and replication-stress signaling activate Ty1 retrotransposition [188], which could promote chromosomal rearrangements involving Ty1. A new Ty1 retrotransposition event at the site of a translocation formed during adaptation to nutrient-limited conditions provides some support for an active role in formation of chromosomal rearrangements [252]. This perspective has led to the suggestion that Ty1 can act as a genome guardian through its various impacts on gene expression, DNA repair, and adaptive genome rearrangements [260]. Comparative experiments manipulating copy numbers and activities of different Ty family members, as well as experiments in other species could help resolve how much of Ty1’s impact on these aspects of genome dynamics are passive effects of a dispersed repeat, general effects of retrotransposon activity, or specific effects due to the particular regulation/activity of Ty1.

There is less literature on the role of Tf1 and Tf2 in chromosomal rearrangements and DNA repair. The Sap1 protein binds LTR sequences and acts as a replication fork barrier [234], as previously noted. The *S. pombe* CENP-B homologs Cbp1/Abp1 and Cbh1 also bind to LTRs and can compete to some extent with Sap1 for binding LTRs [208, 234]. In the absence of CENP-B homologs, replication forks often collapse at LTRs, leading to double-stranded breaks and triggering homologous recombination, indicating that Cbp1/Abp1 and Cbh1 normally stabilize replication forks and restrict recombination at these sites [234]. Whether or not the presence of retrotransposon sequences near replication fork barriers provides an advantage for repair/restart of replication forks, as considered for Ty1, is not clear. While the observations reviewed in this section support some similarities between the impacts of Ty1 and Tf1, each element appears to have distinct relationships with its host.

## Conclusions

Many studies of functional aspects of TEs will be required to develop a sophisticated understanding of their impacts on host organisms. Comparative functional studies offer advantages for isolating and defining crucial mechanisms underlying the impacts of TEs by examining host-TE interactions in diverse contexts. I think that additional characterization of TEs in the two pathogenic yeasts would develop this set of four diverse yeast species into an outstanding model for comparative functional studies of TEs. *Candida albicans* has been recently noted as an emerging model for studies of genome dynamics, partly because of the substantial genetic variation that can be present in this species due to its parasexual cycle [261]. I suggest that TEs be included as an important part of these future genome dynamics studies. Parallel and collaborative studies hold great promise for unraveling the complex layers of TE biology.

## Data Availability

Data sharing not applicable to this article as no datasets were generated or analyzed during the preparation of this review article.

## Abbreviations

*C. albicans*: *Candida albicans*
CNC: Copy number control
*C. neoformans*: *Cryptococcus neoformans*
EN: Endonuclease
IN: Integrase
LTR: Long terminal repeat
NPC: Nuclear pore complex
ORF: Open reading frame
PBS: Primer binding site
PPT: Polypurine tract
PR: Protease
RH: RNase H
RNAi: RNA interference
RT: Reverse transcriptase
*S. cerevisiae*: *Saccharomyces cerevisiae*
*S. pombe*: *Schizosaccharomyces pombe*
siRNA: Short interfering RNA
TE: Transposable element
UTR: Untranslated region
VLP: Virus-like particle
